# Safety Status Monitoring of Operational Rock Bolts in Mining Roadways Under Mining-Induced Effects

**DOI:** 10.3390/s25113486

**Published:** 2025-05-31

**Authors:** Jianjun Dong, Wenduo Ding, Yu Qin, Ke Gao

**Affiliations:** 1 College of Safety Science and Engineering, Liaoning Technical University, Huludao 125105, China; dingwenduo2002@163.com (W.D.); qiny0611@163.com (Y.Q.); gaoke@lntu.edu.cn (K.G.); 2 Key Laboratory of Mine Thermodynamic Disasters and Control of Ministry of Education, Liaoning Technical University, Huludao 125105, China

**Keywords:** mining-induced effects, mining roadways, rock bolts, safety state monitoring

## Abstract

This study focuses on the importance of the real-time monitoring of bolt loads in roadways affected by high-intensity mining and the limitations of conventional monitoring methods. Fiber Bragg grating (FBG) sensors were embedded and encapsulated in rock bolts, and tensile tests were conducted indoors to verify their feasibility. The research was conducted using the consolidated face of the Bultai Coal Mine in the Shendong Mining Area as the engineering background. Real-time monitoring wavelength data from the FBG bolt sensor were obtained through field tests. The analysis of the data aimed to assess the condition of the FBG sensor and variations in axial force within the service bolts of the mining roadway. Using these monitoring results, the real-time stability and safety of the roadway bolts were evaluated. The study indicates that as the working face advances, the axial force in the bolt progressively rises under the effect of mine pressure. The left gang bolt rod’s shaft force changes significantly, while the right gang’s change is relatively small. When the working face moves 60 m past the bolt rod, the axial force in the bolt rises sharply. Moreover, the axial force at different positions of the left and right gang bolts exhibits a distinct variation pattern. The real-time monitoring of bolt support in the return roadway provides essential data for assessing bolt safety.

## 1. Introduction

The necessary condition for the safe production of a mine is to ensure the stability of the return mining roadway [1]. The effective monitoring of stress and strain in the support bolts of the back-mining roadway enhances stability assessment, while in terms of the stress distribution of bolts at different positions within the coal seam affected by mining activities, the characteristics of bolt support are analyzed to help prevent slice gang topping in the back-mining roadway [2].

Yanhui Li et al. investigated the damage mechanism of surrounding rock through the application of the self-stabilized balanced arch theory [3]. Feng Cui et al. analyzed the rupture behavior of the roof plate in the inclined direction at the long-wall working face and examined the stress and energy characteristics of the working face [4]. Housheng Jia et al. revealed the rupture development characteristics and deformation mechanism of the composite roof plate by mainly using the on-site roof probing and numerical simulation [5]. Guodong Li et al. employed digital image correlation (DIC) technology to analyze rock layer deformation and fissure development during coal seam excavation, while pressure sensors and strain gauges were used to monitor mining-induced stress [6]. Chengxing Zhao et al. proposed a composite support method that integrates “injection structure, grouted anchor bolts, grouted anchor cables, and anchor cables” with full-section layered double-arch coordinated reinforcement technology. They validated the reasonableness of this technology through numerical simulations, which demonstrated that the composite support scheme effectively controls deep soft rock tunnels [7]. Juncai Cao et al. proposed and studied a superbolt support scheme. The superbolt support scheme was compared with two other schemes through physical model tests, which demonstrated that the use of super-prestressed bolts could address challenging support problems [8]. Zhe He et al. proposed the theory of bolted grouting support with a fixed bolt length and introduced hierarchical cooperative control technology (HCCT). High-strength hollow grouting ropes and related anchoring methods were studied and evaluated, effectively resolving the long-standing conflict between grouting support and pressure deformation [9]. Jiong Wang et al. proposed a new combined support scheme centered on NPR bolts. The mechanical properties of rocks anchored by NPR bolts were analyzed. Numerical simulations and field tests showed that the combined support scheme successfully mitigates large deformations in soft rock tunnels [10]. Ang Li et al. proposed a bolt reinforcement support design for the deformed and anchored lateral section of the coal pillar. This design proved highly effective in controlling convergent deformation on the lateral side, ensuring safety during roadway excavation and mining [11]. Weijian Yu et al. conducted a field study demonstrating that inclined bolt anchoring across the coal–rock interface in the stress compression zone effectively suppresses uneven deformation in coal–rock composite roadways [12]. The damage to the coal mine roadway is mainly due to the failure of the roadway support role, so the study of the roadway support bolt support effect is an effective means to ensure roadway safety.

Ao Wang et al. examined the stress distribution in regions where fractured rock bolts are located and investigated the mechanism behind bolt breakage [13]. Weijian Yu et al. investigated the causes of failure in rock bolts and cables subjected to repeated disturbances in the return mining roadway [14]. Chen Li et al. designed the support parameters for rock bolts (and cables) by considering the morphological evolution in the plastic zone, thereby ensuring the stability of the roadway [15]. Pu Wang et al. employed a generalized discrete element numerical simulation method to study the stress evolution at the working face and the horizontal distance between the bottom of the roadway. They also analyzed the stability of the roadway bottom and validated their numerical simulation results through field tests measuring the deformation of the surrounding rock and bolt pull-out [16]. Wenyong Wang et al. explored a depth of more than 1000 m to observe tunnel deformation in the first mining period, as well as the distribution of stresses and the effect of bolt support. The finite difference method was used in conjunction with other techniques to analyze the stress distribution and deformation characteristics of the entire working face [17]. Guangkan Jia et al. employed numerical simulations and field tests to investigate the stress evolution of the roadway and analyzed the deformation of the surrounding rock reinforced by bolts [18]. Yanlong Chen et al. conducted numerous field tests to examine the changes in bolt shaft force as the working face advanced through areas affected by mining-induced movement, using the non-destructive testing technique for bolt shaft force, and found that the changes in the bolt shaft force and the distribution of overhanging bearing pressure in the quarry had an obvious corresponding relationship [19]. Xiaohu Liu et al. combined the damage observed in indoor pull-out specimens with the development of the plastic zone and established mechanical models for shear slip damage and shear expansion slip damage at the first anchorage interface based on the elastic–plasticity theory. The relationship between surrounding rock stress and the mechanical properties of anchoring materials was established [20]. Yunhao Wu et al. used numerical simulations and field tests to study the damage characteristics of anchor fractures in deep mining roadways. They quantitatively characterized the deformation pressure of the surrounding rock and the dynamic load on the overburden fractures and established a mechanical criterion for anchor fracture [21].

Therefore, the deployment of distributed fiber optic sensors is a crucial method for monitoring the safety status of roadway support bolts in coal mine production areas. Currently, FBG (Fiber Bragg Grating), BOTDA (Brillouin optical time domain analysis), and OFDR (optical frequency domain reflectometry) represent the three primary distributed fiber optic sensing technologies. BOTDA technology requires long-distance optical fiber deployment and introduces system complexity. Although OFDR provides ultra-high precision and millimeter-level resolution, it is often excessive for roadway monitoring. In contrast, bolt force deformation is primarily concentrated at the anchoring end and free section, making FBG’s point-based measurement ideal. It can be easily affixed to critical points, either through pre-embedding or surface mounting, offering convenient installation. Additionally, FBG sensors are compact, easy to measure, highly integrable, and provide accurate measurements [22,23,24]. They are also resistant to light intensity fluctuations and fiber bending losses, making them widely used for monitoring the safety status of bolts.

**Figure 1:**
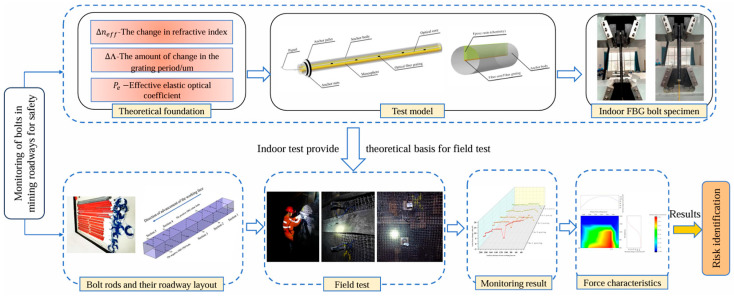
Analysis of safety status of roadway bolts.

However, current research encounters several limitations. FBG sensors mounted on the surface of bolts are prone to detachment under tangential stress, hindering their ability to synchronize with the deformation of the bolt. Furthermore, sensors positioned at the bolt’s end are limited to measuring axial force variations solely at the bolt tray. Meanwhile, the bolt body remains unmonitored, creating a blind spot that prevents real-time observation of its distributed stress–strain condition. In order to effectively solve the above deficiencies, this study buries and encapsulates the fiber grating FBG sensor into the bolt by pulling a slot on one side of the bolt. Tensile tests are carried out indoors to verify their feasibility. The safety condition of the roadway support rock bolts in the back-mining roadway is assessed based on the monitoring data from the on-site test. This study assesses the safety condition of the rock bolts in the back-hoe roadway under the influence of mining (Figure 1) and offers valuable insights into addressing the safety issues of the roadway.

## 2. Monitoring of Sensor Arrangement Schemes

Combined with on-site engineering practice, the engineering application of a fiber grating bolt stress–strain dynamic real-time monitoring system is carried out to assess the actual support effect of bolts during the service period and to offer a theoretical foundation for the design and optimization of the bolt support system.

### 2.1. Basic Situation of Working Surface

The 42203 working faces of Bultai Coal Mine in the Shandong Mining District are arranged according to the direction of inclination of the coal seam. Mining operations are conducted along the strike direction of the coal seam, with the working face extending 320 m in inclined length and 4485.2 m in strike length. The elevation of the ground surface varies between +1251.9 m and +1346.7 m, while the elevation of the bottom plate ranges from 897.42 m to 948.74 m. In the back-mining section, the coal seam lies at depths ranging from 390 to 410 m, with a thickness of 5.6 to 6.2 m and an inclination angle of 1° to 3°. The face is arranged in three roadways, namely the return air roadway, the transportation roadway, and the auxiliary transportation roadway. Among these, the auxiliary transportation roadway of the 42203 working face is significantly influenced by mining activities. Therefore, the 42203 auxiliary transportation roadway is designated as the test section, with a 225 m stretch in front of the selected back-mining working face chosen as the representative test area.

### 2.2. Monitoring Sensor Arrangement

Embedded in the surrounding rock, FBG bolt sensors are employed by the fiber grating monitoring system to measure stress and strain in the rock mass. Through the analysis of real-time monitoring data, the system evaluates the stress and deformation conditions of both the surrounding rock and the bolt support structure, offering early warnings and assessing their stability and safety. The monitoring data obtained by the monitoring system can be transmitted to the ground data acquisition control platform through the fiber optic cable ring network in the tunnel for real-time monitoring. To prevent sensor damage during bolt installation and to effectively monitor the force and deformation of the tunnel bolt support structure in coal mines, the sensor is embedded into the bolt encapsulation and installed in the coal rock alongside the bolt.

The purpose of arranging FBG bolt sensors is to identify the likely locations of bolt rod failures through the statistical analysis of actual bolt breakage occurrences underground. This allows for the determination of the monitoring range and the number of fiber grating sensors required for both the test section and the working face verification test.

## 3. FBG Bolt Design and Installation

### 3.1. FBG Bolt Sensor Working Principle

The grating of the FBG bolt sensor reflects light emitted by a broadband source, and this light is directed through a fiber optic coupler. Subsequently, a mediator device detects the wavelength shift.

FBG satisfies the Bragg condition:

(1) $$\lambda_{B}=2n_{eff}\Lambda$$

where $\lambda_{B}$ is the wavelength of the reflected light center (nm), $n_{eff}$ the effective refractive index, and Λ is the grating period (μm).

The variation relationship between the FBG sensor temperature strain ε and $\lambda_{B}$ is as follows:

(2) $$∆\lambda_{B}=\lambda_{B}\left\{1-\frac{n_{eff}^{2}}{2}\left[p_{12}-\nu \left(p_{11}+p_{12}\right)\right]\right\}\varepsilon +\lambda_{B}\left(\alpha +\xi \right)∆T$$

where $p_{\left[i,j\right]}$ is the Pockels coefficient, $∆\lambda_{B}$ is the grating wavelength drift, ε is the strain, $n_{eff}$ is the effective refractive index, α is the thermal expansion coefficient, ν is Poisson’s ratio, ξ is the thermal optical coefficient, and ΔT is the temperature change (°C).

### 3.2. Fiber Grating Strain Measurement

When the fiber grating is subjected to stress only, it is

(3) $$\frac{\Delta \lambda_{B}}{\lambda_{B}}=\frac{\Delta \Lambda }{\Lambda }+\frac{\Delta n_{eff}}{n_{eff}}$$

where $\Delta n_{eff}$ is the amount of change in the refractive index and ΔΛ is the amount of change in the grating period.

In a constant temperature environment, the fiber grating strain is measured by Eq:

(4) $$\varepsilon =\frac{\Delta \lambda_{B}}{\lambda_{B}}\frac{1}{1-p_{e}}=1.28\frac{\Delta \lambda_{B}}{\lambda_{B}}$$

where $p_{e}$ is the effective elasticity coefficient, and it has a value of 0.22.

### 3.3. FBG Bolt Design

**Figure 2:**
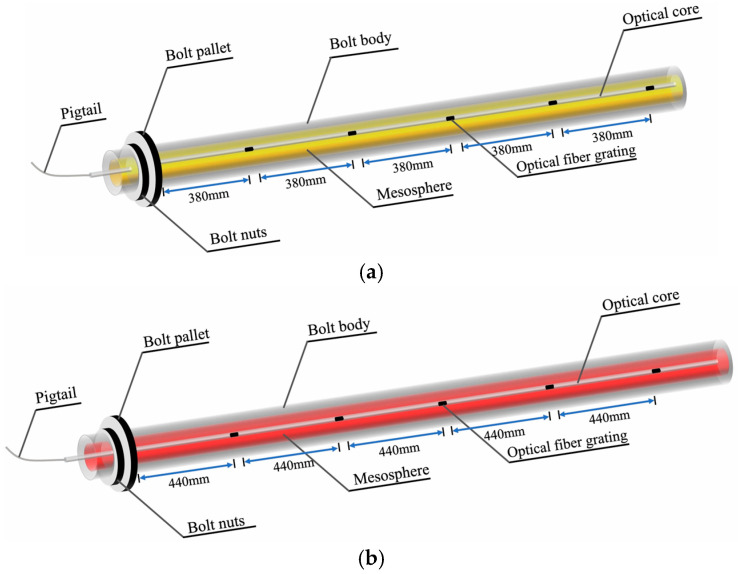
Structural arrangement of bolt FBG sensors. (**a**) Structural arrangement of rebar bolt FBG sensors; (**b**) structural arrangement of FRP bolt FBG sensors.

An 18 mm diameter rebar bolt and 32 mm diameter FRP (fiber-reinforced polymer) bolt were processed to be 2 mm wide, with a depth of 9 mm and a 16 mm minor groove. The FBG sensor was firmly fixed at the base of the groove, encapsulated within the bolt. The armored fiber optic cable was linked to the FBG modem instrument at its end, enabling real-time monitoring. The FBG sensors were sequentially arranged and numbered at intervals of 380 mm on a 2.1 m rebar bolt and 440 mm on a 2.4 m FRP bolt, respectively. The structural arrangement of the bolt FBG sensor is shown in Figure 2.

**Figure 3:**
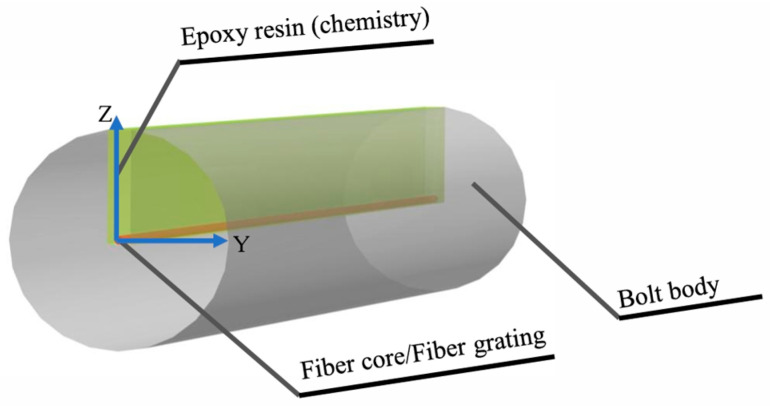
Schematic of the FBG sensor after it is buried in the structure.

The cross-sectional view of the FBG sensor is depicted in Figure 3. The FBG sensor was composed of three components, namely the bolt rod body, epoxy resin, and the FBG.

**Figure 4:**
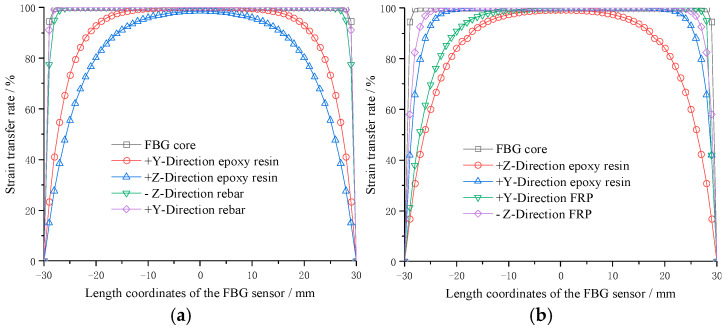
The relationship curve between the strain transfer rate of the FRP matrix and length coordinate. (**a**) Rebar; (**b**) FRP.

The shear moduli of elasticity of the rebar and FRP substrates were identical in all directions. The strain transfer rate at each point within the FBG sensor’s scale range is presented in Figure 4. The horizontal axis in Figure 4 denotes the distance from the sensor’s center point, whereas the vertical axis displays the directional strain transfer rates for different materials. Analysis shows that for both materials, the strain transfer rate exhibits a symmetrical decline from the center outward, eventually diminishing to zero at the extremities. The rate is highest at the center point and gradually decreases as it moves away from the center, with the rate of change being slower near the center compared to farther positions. Within the 10 mm grid region (spanning −5 mm to 5 mm), the intermaterial strain transfer loss remains negligible. Therefore, FBG wavelength variations provide precise representations of bolt stress conditions.

### 3.4. Incremental Modulus of Elasticity of Bolt

The axial force of the bolt can be expressed as

(5) $$F=AE\varepsilon =1.28EA\frac{\Delta \lambda_{B}}{\lambda_{B}}$$

where F is the bolt axial force (N), e is the modulus of elasticity of the bolt (MPa), and A is the bolt cross-sectional area (m^2^).

When the axial force on the bolt is small, the resulting change in its cross-sectional area is minimal and insignificant. Therefore, it can be considered as a constant. The modulus of elasticity of the bolt is constant after the material of the bolt is determined.

**Figure 5:**
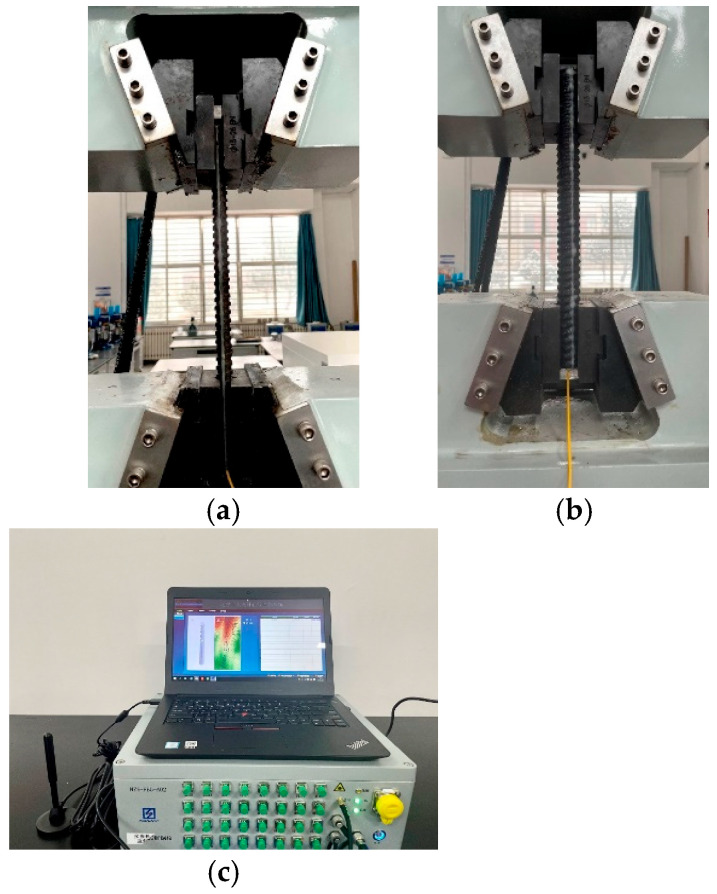
Bolt tensile tests. (**a**) Rebar; (**b**) FRP; (**c**) FBG demodulation platform.

**Figure 6:**
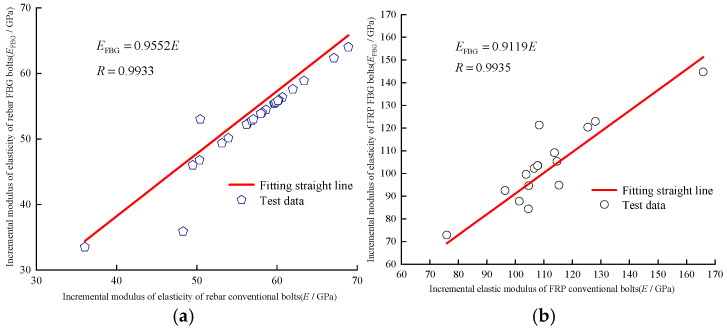
Relationship curve of incremental modulus of elasticity of bolts. (**a**) Rebar; (**b**) FRP.

Conventional FRP bolts, threaded steel bolts, FBG FRP bolts, and FBG rebar bolts were individually stretched at a constant temperature and speed using the electro-hydraulic servo universal testing machine, as shown in Figure 5. From the experimentally measured FBG sensor center wavelength change data, Figure 6 illustrates the relationship curves of the incremental elastic modulus for both conventional and FBG rock bolts.

The incremental modulus of elasticity of the bolt is defined during the elastic deformation stage as the ratio of the change in axial force to the corresponding change in strain, which occurs between the stretching and yielding phases. The test data shown in Figure 6 reveal that the incremental modulus of elasticity for both rebar conventional bolts and FBG bolts is consistent, which is in line with the mechanical property that the modulus of elasticity remains constant during the elastic deformation stage. The average incremental moduli of elasticity for rebar conventional bolts and FBG bolts are 54.2 GPa and 50.4 GPa, respectively. The average incremental moduli of elasticity for FRP conventional bolts and FBG bolts are 112.2 GPa and 107.7 GPa, respectively. The strength loss aligns with the practical requirements of underground conditions. The experimental results further validated the reliability of FBG bolt sensors in monitoring the axial force of the bolts.

### 3.5. Monitoring Sensor Installation

The bolt damage induced by roof cycle pressure is primarily concentrated in the two sections of the transportation and auxiliary transportation lanes.

**Figure 7:**
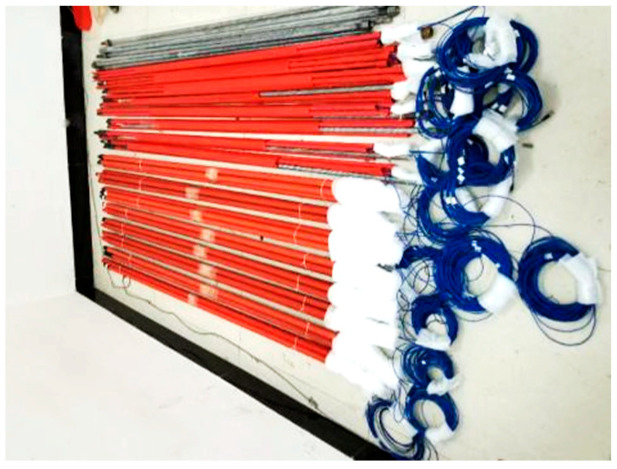
FBG bolts.

**Figure 8:**
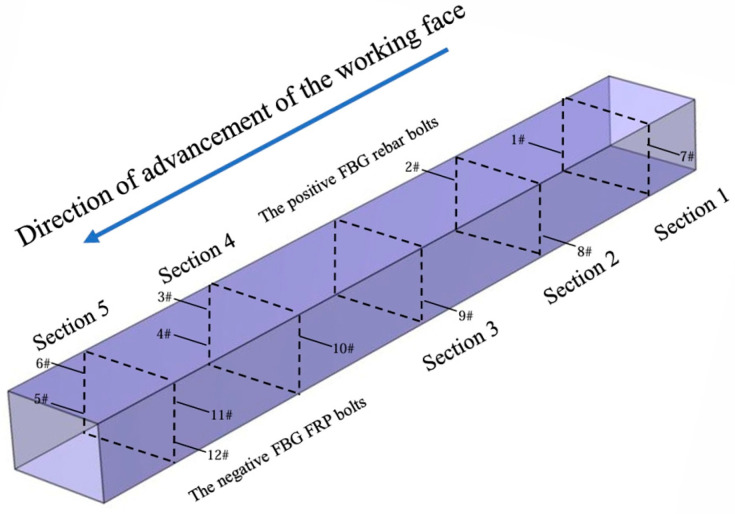
Bolt installation position.

The maximum step length of the working face is 15 m to monitor the deformation of the roadway support bolts within the range of cyclic pressure and the law between the working face advancing process and the mine pressure, that is, the fiber optic grating sensor bolts need to be arranged in front of the working face within a range of about 225 m. To safeguard the sensor from potential damage during transit, a thermoplastic tube was employed for encapsulation. As illustrated in Figure 7, both the bolt connection and the pigtail were encased in plastic foam, providing moisture resistance and cushioning to protect the sensor during transportation or when moved to the underground mine. A total of twelve bolts were installed in the roadway perimeter rock, with six FRP bolts placed in the positive gang and six rebar bolts arranged in the negative gang, as shown in Figure 8.

Positive and negative gang side were due to the existence of interference from underground facilities, according to the site, to make adjustments to the positive and negative gang fiber grating bolt positions. Monitoring the force condition of the surrounding rock roadway was a prolonged and gradual process. To improve the accuracy of the monitoring data, it was essential to conduct daily trips down the well, record real-time conditions, maintain consistency in the external environment, and accurately collect the bolt data.

**Figure 9:**
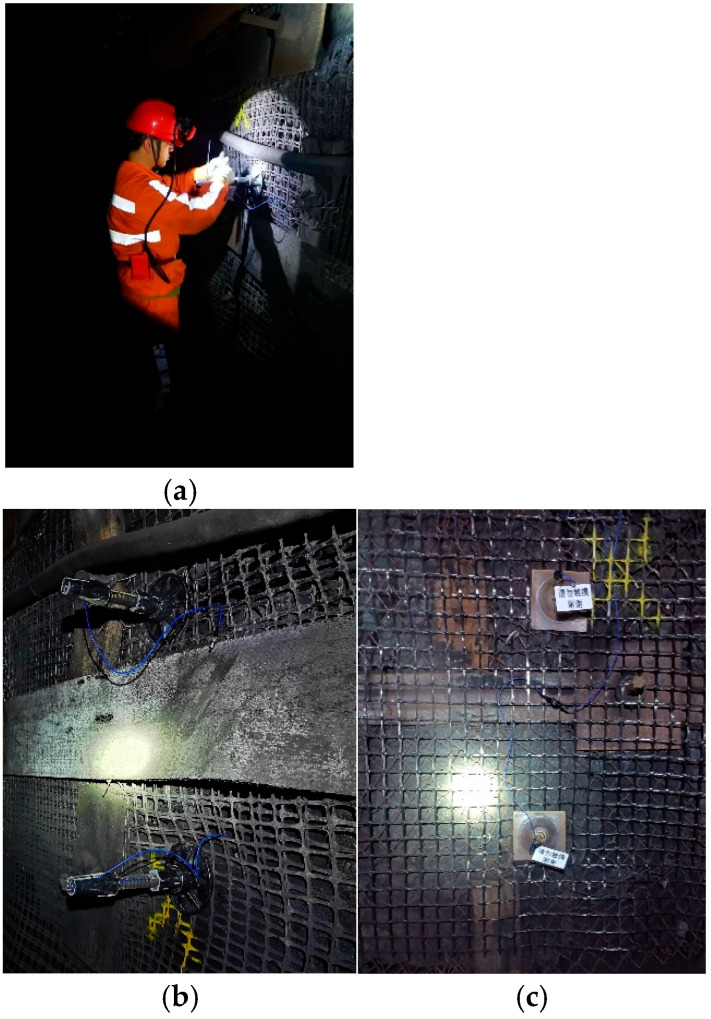
FBG bolt sensor field tests. (**a**) The installation of FBG bolts. (**b**) The positive FBG FRP bolts. (**c**) The negative FBG rebar bolts.

The bolts were manually driven using hand-held drilling, the bolting resin was mixed with a stirring bolt post-drilling, and the fiber optic grating bolts were subsequently driven in. After the rock bolts were driven into the roadway, each gang side bolt rod was connected with the model MGTS-12B mining single-mode fiber optic cable, and at the same time, the mining fiber optic cable was installed near the demodulator and routed to the hub box. Figure 9 illustrates the test setup of the FBG bolt sensor in the field roadway.

## 4. Bolt Rod Body Force and Deformation Characteristics

### 4.1. Analysis of Monitoring Results

The relatively constant temperature of the underground tunnel ensures stability after the rock bolts are embedded in the surrounding rock, with minimal impact on the sensors.

**Figure 10:**
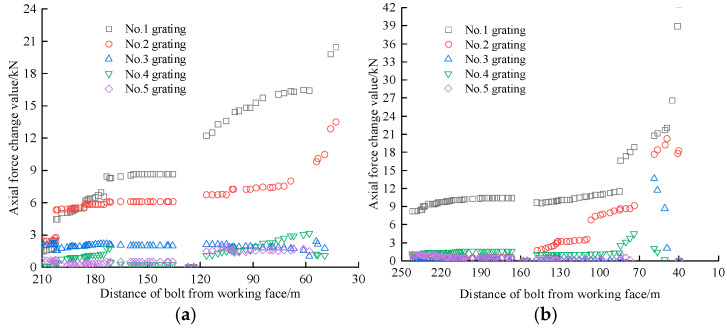

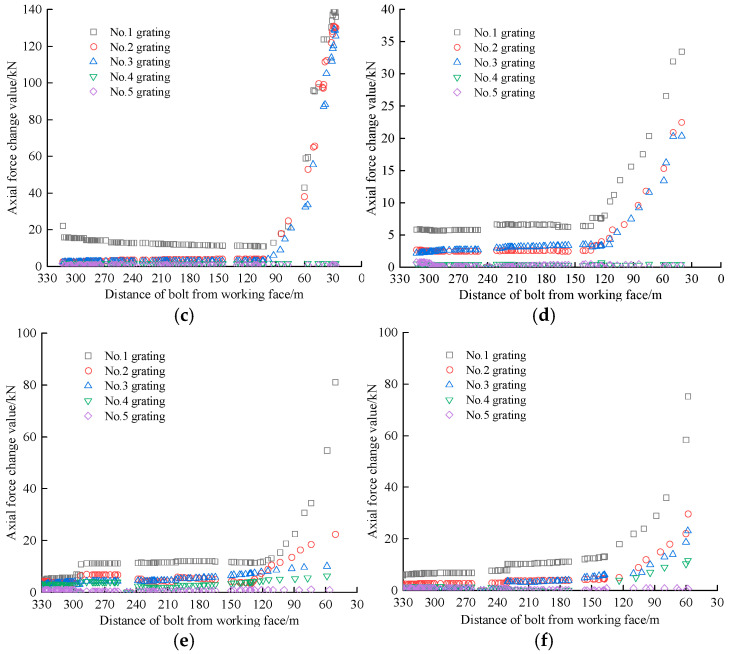
Positive gang bolt axial stress monitoring test curve. (**a**) Bolt 1. (**b**) Bolt 2. (**c**) Bolt 3. (**d**) Bolt 4. (**e**) Bolt 5. (**f**) Bolt 6.

On the positive and negative gang bolts, the FBG sensors were arranged and numbered sequentially at a spacing of 440 mm and 380 mm, respectively. For ease of analysis, the sensors were numbered from the exposed end to the bolted end, with grating No. 1 for the pallet section and grating No. 5 for the bolted section. The data were collected until the failure of bolt 1 so that the safety status of each bolt could be compared and analyzed. The FBG sensor accurately reflects the axial force at each position of the bolt bar as the working face advances, with the test curve of the axial stress monitoring for the positive gang bolt bar shown in Figure 10.

Figure 10 illustrates that the axial stress distribution along the positive gang bolt remains consistent overall, while the axial force exhibits notable variations across the bolt body. Notably, the No. 1 grating displays the most pronounced variations, exhibiting faster data accumulation rates than the No. 2–5 grating located at deeper burial depths. These findings suggest that grating sensors positioned closer to the pallet demonstrate superior axial force monitoring efficacy compared to their counterparts in the anchored section, consequently registering more substantial force variations.

As illustrated in Figure 10b,c, the axial force evolution can be categorized into three distinct phases, namely slow growth, rapid growth, and yielding. Initially, the bolt’s axial force exhibits a gradual increase when the working face is beyond 120 m. Subsequently, a sharp rise occurs within the 30–120 m range, followed by a transition to the yielding state when the working face approaches within 30 m.

Of the six fiber grating bolts installed in the main gang, only bolts 2 and 3 have reached the yielding state. Bolt 2 is positioned at the 2701 m mark in the roadway, situated 2.3 m below the top plate, and experiences a No. 1 grating yielding axial force of 108 kN. Bolt 3 is situated at the 2773 m position in the roadway, also 2.3 m below the top plate, with yielding axial forces of 136.15 kN, 130.94 kN, and 129.21 kN for the No. 1, No. 2, and No. 3 gratings, respectively. These two bolts are spaced 72 m apart. As specified by underground safety standards, the pull-out force for any underground bolt must exceed 80 kN. The FBG bolt sensor monitoring system fully satisfies the operational requirements of the mine. Although bolts 5 and 6 did not enter the yielding state, their axial forces experienced notable changes, increasing by approximately 80 kN. Consequently, the induced stress on the bolts surpassed the mine’s designed prestress levels, indicating potential damage. While the axial force variations in bolts 1 and 4 are relatively minor, their potential risks should not be underestimated. Close monitoring is required to observe any progression towards a damaged state, allowing for timely intervention if necessary.

**Figure 11:**
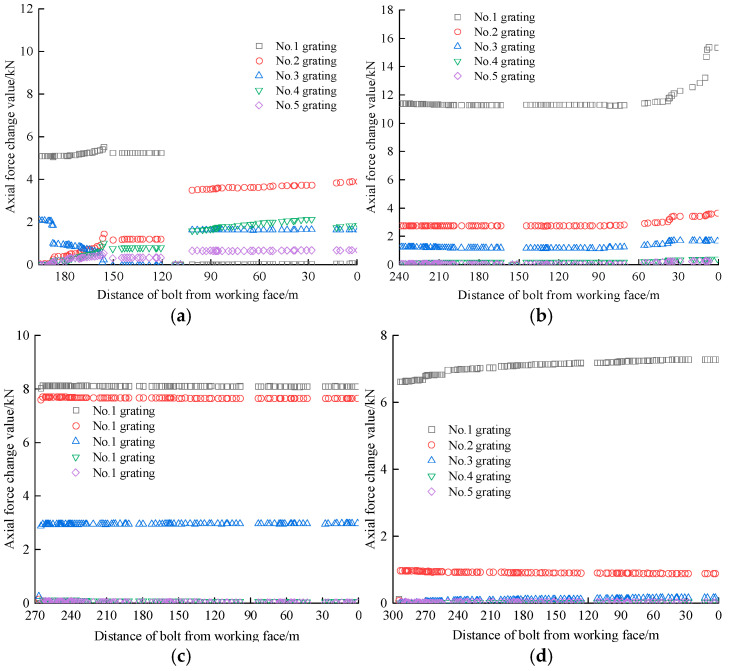

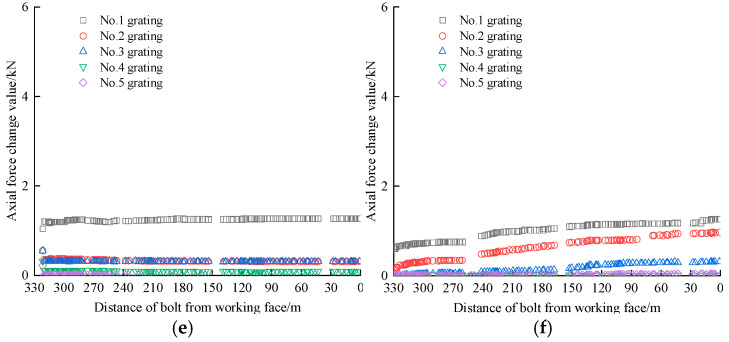
Negative gang bolt axial stress monitoring test curve. (**a**) Bolt 7. (**b**) Bolt 8. (**c**) Bolt 9. (**d**) Bolt 10. (**e**) Bolt 11. (**f**) Bolt 12.

Figure 11 illustrates the axial stress monitoring curve for the negative gang bolt. As shown in Figure 11, the overall trend of axial stress variation in the negative gang bolt remains consistent. However, the axial force exhibits only minor variations as the working face advances. This trend intensifies due to the effect of the No. 1 grating on the free section of the bolt, where the initial data are influenced by deformation, leading to a higher axial force in the bolt. The axial force recorded by the grating sensors near the tray in rock bolts No. 7, 8, 9, and 10 is greater than that in bolts 11 and 12. Compared to these, the grating sensors positioned near the tray register a higher axial force. The axial force distribution of the bolt mirrors that of the positive gang bolt. Additionally, as the grating approaches the pallet, both the monitored axial force and its variation increase.

In the negative gang, the axial force growth of the six FBG bolt sensors remains smooth, with no bolt yielding or rapid axial force increases observed. An exception was observed in bolt 8, which demonstrated a rapid force increase when the working face approached within 30 m. Upon reaching the bolt position, the working face induced maximum axial force increments of 15.32 kN and 8 kN in the primary gratings of bolts 8 and 9, respectively. These measurements suggest the bolts entered an anomalous stress state, necessitating continuous monitoring of their force variations.

### 4.2. Bolt Rod Body Force Characteristics

**Figure 12:**
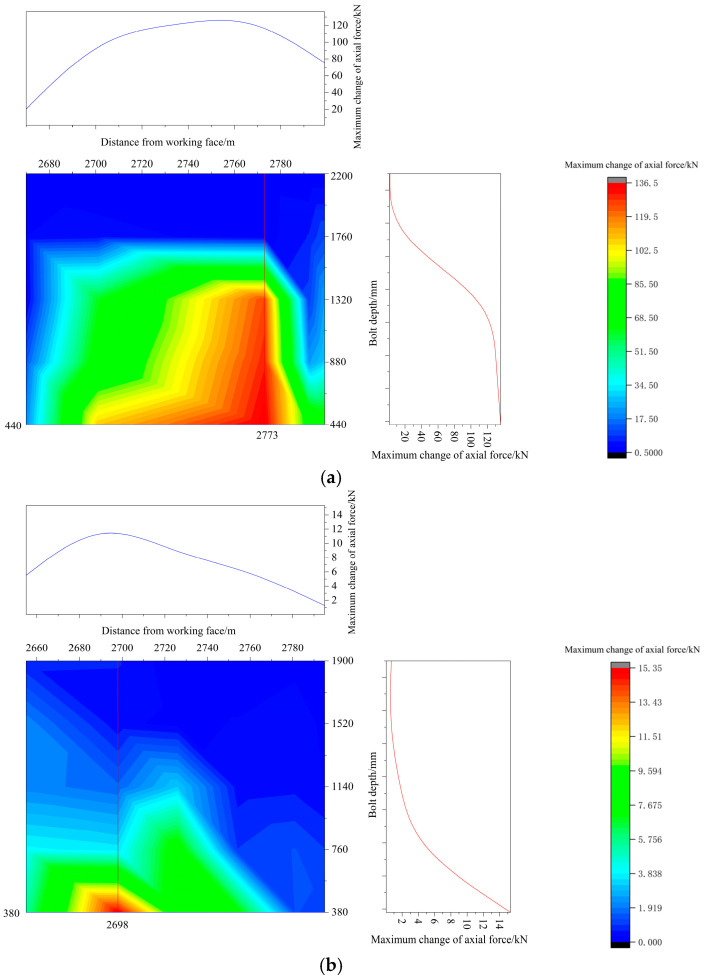
Bolt monitoring data contour plots and profile curves. (**a**) Positive gang bolt monitoring data contour plot and profile curve. (**b**) Negative gang bolt monitoring data contour plot and profile curve.

Using the bolt monitoring data, a continuous distribution contour profile curve can be generated through interpolation and smoothing, as illustrated in Figure 12.

As illustrated in Figure 12, the axial force in the rock bolts peaks in both parallel and perpendicular directions to the mining face. The peak axial force occurs between 2680 and 2720 m in the positive gang of the mining roadway. Simultaneously, the negative gang of the mining roadway also experiences the highest axial force within this range, posing the greatest risk of roof damage to the surrounding rock. Special attention is needed for this roadway position when the mining face is being mined back. The positive gang of the roadway 40 m in front of the workings adopts over-advanced support to prevent the two gangs from being damaged when the auxiliary transportation lane is mined back, which leads to part of the mining pressure being borne by it; this leads to a greater disparity in the axial force between the two roadway gangs.

In the direction of the mining face, the positive gang bolt is embedded at depths ranging from 0 to 440 mm, while the negative gang bolt is installed at depths of 0 to 380 mm. These depth ranges mainly form the free section, permitting unrestricted deformation, which in turn leads to considerable variations in axial force. With increasing bolt embedment depth, the peak axial force declines. This occurs because a deeper embedded bolt integrates more effectively with the surrounding rock and moves in tandem with its deformation. Thus, the change in the axial force is less. Both deep and shallow rock bolts are located in the free section, close to the buttresses, allowing them to deform freely, which results in more significant changes in axial forces. The axial force change in the positive gang bolt is more pronounced than in the negative gang bolt, with the change in axial force generally being larger in the positive gang bolt. The gang’s proximity to the working face results in it being the first to be affected by mining, making its influence more direct compared to other locations.

### 4.3. Evaluation of the Safety Status of Bolts

As the working face progresses, the maximum axial force at each bolt position steadily increases. However, due to multiple factors during the advancement process, data discontinuities occur, and some measurements are only captured at the center wavelength when the face nears the bolt. During the working face’s progression, only bolts 2 and 3 exhibit a rapid rise in axial force, eventually reaching the yield state. The maximum changes in axial force are presented in Table 1.

In accordance with the coal mine roadway bolt support standard GB/T 35056-2018, bolts are categorized into three distinct states, namely normal, abnormal, and destroyed. Table 2 illustrates the safety state evaluation results for the back-mining roadway bolts.

## 5. Conclusions

(1) In the axial stress monitoring test using FBG sensors at the coal mine site, the positive gang bolt bar exhibits the most significant change in axial force. The variation in axial force is generally greater than that observed in the negative gang bolt bar. The roadway gang closest to the working face is subjected to the shortest distance and the most direct impact from mining activities.
(2) As the comprehensive mining face advances, the positive gang bolt rod maintains a 60 m distance from the working face. Beyond this distance, the axial force on the bolt rod starts to rise progressively. Once the working face advances 60 m past the bolt rod, its axial force experiences a significant rise. With the advancement of the working face, the axial force on the negative gang bolt rod steadily rises.
(3) Based on the monitoring data of the axial force in the rock bolts within the underground tunnel, the magnitude of the change in the shaft force in the bolted section is small because the rods are relatively fit to the surrounding rock; the rods in the near-pallet area are situated in the free section, allowing them to deform freely, resulting in a significant change in axial force; in the free section, the axial force along the rock bolts is unevenly distributed, with the highest force occurring in the rods nearest to the pallet area.
(4) By interpolating and smoothing the monitoring data of FBG rock bolts at the working face, a continuous contour profile curve distribution is generated. By comparing the distribution of maximum axial force values along the rock bolts at different positions, it is possible to identify the roadway areas most influenced by mining activities. This enables the implementation of timely bolt support and preventive measures to avoid accidents such as broken rock bolts.

## Figures and Tables

**Table 1:** Statistics of the maximum value of the axial force of the bolt.

Bolt Number	Maximum Value of Change in Bolt Axial Force/kN	Ratio of Bolt Axial Force to Design Anchorage Force/%
Bolt 1	20.44	76.04
Bolt 2	108.11	>100
Bolt 3	136.15	>100
Bolt 4	33.42	90.47
Bolt 5	81.13	>100
Bolt 6	75.29	>100
Bolt 7	5.52	59.47
Bolt 8	15.32	70.36
Bolt 9	8.09	61.32
Bolt 10	7.27	59.41
Bolt 11	1.27	54.74
Bolt 12	1.25	54.72

**Table 2:** Bolt safety status evaluation statistics.

Bolt Status	Condition	Bolt Number
Normal	Bolt axial force less than 60% of design anchorage force	Bolt 7, Bolt 10, Bolt 11, Bolt 12
Abnormal	Bolt axial force is 60–100% of design anchorage force	Bolt 1, Bolt 4, Bolt 8, Bolt 9
Destroyed	Bolt axial force greater than design anchorage force	Bolt 2, Bolt 3, Bolt 5, Bolt 6

## Data Availability

All data and code used or analyzed in this study are available from the corresponding author upon reasonable request.
